# Dr. E. D. Leffingwell

**Published:** 1916-10

**Authors:** 


### OBITUARY

Readers are requested to report promptly the death of all physicians in Western New York, or former residents of this region, or graduates of any medical school in Western New York, and to notify the families of the deceased of our desire to publish adequate Obituary notices.

---

**Dr. E. D. Leffingwell,** brother of the above, Bellevue Hospital Medical College, 1877, died at his home in Oswego, N. Y., Sept. 10, aged 67. In 1881, after the destruction by fire of the old Dansville Sanitarium, and during the construction of the present edifice of the same (now the Jackson Health Resort), he joined the medical staff and continued in the service for some years.

